# Cytomegalovirus infection reduced CD70 expression, signaling and expansion of viral specific memory CD8^+^ T cells in healthy human adults

**DOI:** 10.1186/s12979-022-00307-7

**Published:** 2022-11-11

**Authors:** Jian Lu, Guobing Chen, Arina Sorokina, Thomas Nguyen, Tonya Wallace, Cuong Nguyen, Christopher Dunn, Stephanie Wang, Samantha Ellis, Guixin Shi, Julia McKelvey, Alexei Sharov, Yu-Tsueng Liu, Jonathan Schneck, Nan-ping Weng

**Affiliations:** 1 Laboratory of Molecular Biology and Immunology, National Institute on Aging, NIH, Baltimore, MD USA; 2 Current address: Division of Microbiology and Immunology, School of Medicine, Jinan University, 601 Huangpu Ave West, Tianhe District, Guangzhou, China; 3 Diagnologix LLC, San Diego, CA USA; 4 Apheresis Unit, Laboratory of Clinical Investigation, National Institute on Aging, NIH, Baltimore, MD USA; 5 Laboratory of Genetics and Genomics, National Institute on Aging, NIH, Baltimore, MD USA; 6 University of California San Diego, La Jolla, CA USA; 7 Department of Pathology, Johns Hopkins University School of Medicine, Baltimore, MD USA

**Keywords:** Chronic infection, CD70, CMV-specific CD8^+^ T cells, Activation, Expansion

## Abstract

**Background:**

Cytomegalovirus (CMV) infection leads to effector memory CD8^+^ T cell expansion and is associated with immune dysfunction in older adults. However, the molecular alterations of CMV-specific CD8^+^ T cells in CMV infected healthy young and middle-aged adults has not been fully characterized.

**Results:**

We compared CD8^+^ T cells specific for a CMV epitope (pp65_495-503_, NLV) and an influenza A virus (IAV) epitope (M1_58-66_, GIL) from the same young and middle-aged healthy adults with serum positive for anti-CMV IgG. Compared to the IAV-specific CD8^+^ T cells, CMV-specific CD8^+^ T cells contained more differentiated effector memory (T_EM_ and T_EMRA_) cells. Isolated CMV-specific central memory (T_CM_) but not naïve (T_N_) cells had a significant reduced activation-induced expansion *in vitro* compared to their IAV-specific counterparts. Furthermore, we found that CD70 expression was reduced in CMV-specific CD28^+^CD8^+^ T_CM_ and that CD70^+^ T_CM_ had better expansion *in vitro* than did CD70^-^ T_CM_. Mechanistically, we showed that CD70 directly enhanced MAPK phosphorylation and CMV-specific CD8^+^ T_CM_ cells had a reduced MAPK signaling upon activation. Lastly, we showed that age did not exacerbate reduced CD70 expression in CMV- specific CD8^+^ T_CM_ cells.

**Conclusion:**

Our findings showed that CMV infection causes mild expansion of CMV-NLV-specific CD8^+^ T cells, reduced CD70 expression and signaling, and proliferation of CMV-NLV-specific CD8^+^ T_CM_ cells in young and middle-aged healthy adults and revealed an age-independent and CMV infection-specific impact on CD8^+^ memory T cells.

**Supplementary Information:**

The online version contains supplementary material available at 10.1186/s12979-022-00307-7.

## Background

CD8^+^ T cells provide essential cell-mediated immunity and surveillance against viral infections and cancerous cells [1, 2]. During the first viral encounter, antigen-specific naïve CD8^+^ (T_N_) T cells are responsible for generating effector cells that contain and eventually eliminate virally infected cells and for differentiating to long-lived memory CD8^+^ T cells [3]. Memory CD8^+^ T cells are capable of rapidly activation and expansion offering a more effective control of subsequent encounters of the same virus compared to the initial T_N_ response [4]. However, when a virus causes chronic or latent infection such as cytomegalovirus (CMV), the memory CD8^+^ T cell response becomes complex as the interaction between viral-specific CD8^+^ T cells and virus is at a continuous state of “checks and balances” [5]. Consequently, chronic or latent CMV infection can eventually lead to the inflation of terminally differentiated memory CD8^+^ T cells and immune dysfunction in old adults [6–8]. Recent study of CMV infection in healthy young and middle-aged adults showed increased CD57 expression in T cells [9], but it is unknown whether such increase only in CMV-specific or in non CMV-specific T cells. Overall, the molecular mechanisms of CMV infection caused changes of CMV-specific CD8^+^ T cells in healthy young and middle-aged adults is not fully examined.

CMV, a herpesvirus (HHV-5), causes life-long infection in humans with an estimated 60-90% of the world population infected. Most immunocompetent hosts carry the virus in the symptom-free latency stage [10]. CMV IgG positivity, an indicator of CMV infection, increases from 36.3% in 6–11-year-olds to 90.8% in those over 80 [11]. CMV infection induces differentiation of CMV-specific CD8^+^ T cells [12], which are responsible for containing CMV reactivation and the life-long interaction with CMV leads to significant expansion of CMV-specific effector memory CD8^+^ T cells in old humans [13]. Although human CMV has the largest genome of any known human virus, encoding over 162 potential proteins [14, 15], the epitopes of CMV recognized by CD8^+^ T cells appear broad and display individual dominance associated with limited HLA haplotype (HLA-B7 and HLA-A2) [16, 17]. CD8^+^ T cells that recognize an epitope of CMV phosphoprotein 65 (pp65_495–503_ or NLVPMVATV) accounted for approximately 20% of total CD8^+^ T cell responses to CMV in HLA-A2^+^ subjects [17, 18]. Analysis of CD8^+^ T cells specific to pp65 shows differentiation and loss of CD28 expression in old humans [19]. However, it is currently unknown when such alterations occur in CMV-specific CD8^+^ T cells in young and middle-aged healthy adults and whether early functional changes exist in these CMV-specific CD8^+^ T cells prior to loss of CD28 and clonal expansion and differentiation.

The activation-induced proliferation of CD8^+^ T cells requires the engagement of the TCR with peptide presented by MHC class I and co-stimulatory receptors such as CD28, CD27, and others [20, 21]. These co-stimulatory receptors interact with the ligand on the surface of antigen-presenting cells to amplify TCR signals and to produce autocrine cytokines such as IL-2 for sustained proliferation of activated CD8^+^ T cells [22]. In old humans who have CMV infection, CMV-specific CD8^+^ T cells are more differentiated and impaired in antigen-induced proliferation due to loss of CD28 and other co-stimulatory receptor expression [6, 23, 24]. The engagement of CD27 and its ligand CD70 has long been recognized as an important co-stimulatory signaling pathway regulating T cell activation and function [25, 26]. Decreased magnitudes of CMV-specific CD4^+^ and CD8^+^ T cell responses in the absence of CD27-CD70 co-stimulation have been reported in a mouse CMV infection model [27], but this has not been demonstrated in human CMV infection. While most studies of CD27-CD70 signaling in T cells view CD70 as just a ligand (expressed on the antigen-presenting cell surface) of CD27 (expressed on T cell surface) signaling, Deola et al. show that B cells expressing CD27 can promote CD8^+^ T cell survival and proliferation through CD70 [28]. Transgenic expression of CD70 increased CD8 T cell expansion in response to antigen challenge *in vivo* in mouse [29]. However, whether engaging CD70 delivers signals in CD70^+^ T cells remains to be determined. A recent report shows that CD70^+^CD8^+^ T cells increase in all four major subsets of T cells with age [30], opposite to the age-related reduction of CD27 in CD8^+^ T cells. Therefore, it is of great interest to elucidate the mechanistic relationship of such opposite changes of CD27 and CD70 expressions in CD8^+^ T cells with aging.

To investigate the changes of CMV-specific CD8^+^ T cells, we compared the phenotype and *in vitro* expansion of CMV pp65_495–503_ (referred to as CMV)- and influenza A virus (IAV) M1_55-64_ (GILGFVFTL) (referred to as IAV)-specific CD8^+^ T cells from the same HLA-A2^+^ and CMV IgG^+^ healthy young and middle-aged adults. We observed that CMV-specific CD8^+^ T cells were more differentiated than IAV-specific CD8^+^ T cells and had less expansion in response to antigen stimulation *in vitro* than IAV-specific CD8^+^ T cells. This reduced expansion was due to a reduced expansion of CMV-specific memory (T_CM_) not CMV-specific T_N_ CD8^+^ T cells. Transcriptome analysis showed that CD70 expression was lower in CMV-specific T_CM_ than in IAV-specific T_CM_ cells. Furthermore, we showed that isolated CD70^+^ T_CM_ proliferated better than CD70^-^ T_CM_ *in vitro* post-anti-CD3 and anti-CD28 (anti-CD3/CD28) stimulation and that activation induced MAPK signaling via increased ERK1/2 phosphorylation was enhanced in CD70 expressed T cells. Reduced CD70 expressed CMV-specific T_CM_ cells exhibited a reduced ERK1/2 phosphorylation compared to IAV-specific T_CM_ upon antigen stimulation *in vitro*. Lastly, advancing age did not exacerbate the reduced CD70 expression in CMV-specific T_CM_ compared to IAV-specific T_CM_ cells. Together, our findings show that CMV infection in healthy young and middle-age adults result in reduced proliferation in CMV-specific CD8^+^ T_CM_ cells due partially to reduced CD70 expression and signaling.

## Results

### Antigen-specific expansion and differentiation of CD8^+^ cells in CMV-infected healthy young and middle-aged adults

**Fig. 1:**
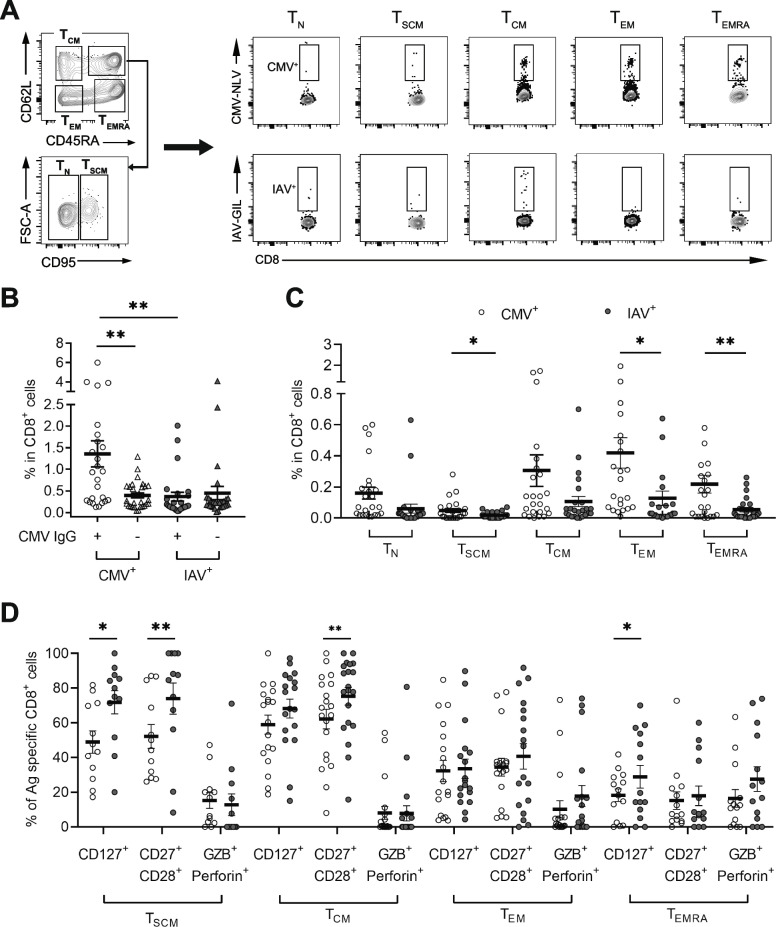
Frequency and differentiation status of IAV- and CMV-specific CD8^+^ T cells in blood of CMV infected healthy adults. **A** Gating of CMV-NLV (pp65_495–503_) or IAV-GIL(M1_58-66_) -specific CD8^+^ T cell subsets. CMV- and IAV-specific cells from the same donor were further classified into T_N_ (CD45RA^+^CD62L^+^CD95^-^), T_SCM_ (CD45RA^+^CD62L^+^CD95^+^), T_CM_ (CD45RA^-^CD62L^+^), T_EM_ (CD45RA^-^CD62L^-^), and T_EMRA_ (CD45RA^+^CD62L^-^). **B** Frequencies of CMV- or IAV-specific CD8^+^ T cells in CMV IgG positive (CMV infected) and negative young and middle-aged (<65-year-old) healthy adults (*N*=18 each, age- and sex-matched). **C** Frequencies of CMV- and IAV-specific CD8^+^ T cell subsets in CMV IgG positive young and middle-aged healthy adults (*N*=25). **D** Percentages of CD127^+^, CD27^+^CD28^+^, and Granzyme B (GZB)^+^ perforin^+^ cells in CMV- specific and IAV-specific CD8^+^ T cell subsets from CMV IgG positive young and middle-aged healthy adults (*N*=20). Data represent the mean values ± standard error of the mean (SEM). * Indicates *P* < 0.05, ** indicates *P* < 0.01

To determine the impact of CMV infection on CD8^+^ T cells, we compared frequency and differentiation of CMV (pp65_495–503_, NLVPMVATV)- and IAV (M1_55-64,_ GILGFVFTL)-specific CD8^+^ T cells from CMV-IgG positive and negative healthy young and middle-aged adults (<65-year-old) by flow cytometry (Fig. 1A). We determined CMV infection by the presence of CMV IgG in serum and found that the frequency of CMV-specific CD8^+^ T cells were significantly increased (14.5 ± 8.1-fold, Mean ± SEM) in CMV IgG positive subjects than in CMV IgG negative subjects (Fig. 1B, Supplemental Table S1). In contrast, the frequency of IAV-specific CD8^+^ T cells was comparable between CMV IgG positive and negative subjects. Within the CMV IgG positive subjects, the frequency of CMV-specific CD8^+^ T cells was also significantly higher (9.5 ± 2.7-fold) than that of IAV-specific CD8^+^ T cells (Fig. 1B). To further determine which subsets of CD8^+^ T cells contribute to the differences between CMV- and IAV-specific CD8^+^ T cells, we compared five subsets: Naive cells (T_N,_ CD45RA^+^CD62L^+^CD95^-^), memory stem cells (T_SCM,_ CD45RA^+^CD62L^+^CD95^+^), central memory cells (T_CM,_ CD45RA^-^CD62L^+^), effector memory cells (T_EM,_ CD45RA^-^CD62L^-^), and effector memory cells re-expressing CD45RA (T_EMRA,_ CD45RA^+^CD62L^-^) of CMV-specific CD8^+^ T cells (Fig. 1A), and found that CMV-specific CD8^+^ T cells had three memory subsets (T_SCM_, T_EM,_ and T_EMRA_) significantly higher than their corresponding IAV-specific memory subsets in CMV IgG positive subjects (Fig. 1C). These findings suggest that healthy young and middle-aged adults infected with CMV have expanded differentiated CMV-specific memory CD8^+^ T cells.

Next, we compared the expression of a panel of different markers in subsets between CMV- and IAV-specific CD8^+^ T cells from the same CMV infected subjects. Compared to IAV-specific CD8^+^ T cell subsets, there was no significant difference in expression of these markers in CMV-specific T_N_ cells (Supplemental Fig. S1A). CMV-specific T_SCM_ and T_EMRA_ cells showed reduced CD127 (IL-7R) expression and CMV-specific T_SCM_ and T_CM_ cells expressed reduced CD27^+^CD28^+^ (Fig. 1D). These findings demonstrate that CMV-NLV specific memory subsets decrease co-stimulatory and growth signals. Collectively, the results suggest that chronic infection drives further differentiation of CMV-specific memory CD8^+^ T cell subsets in healthy young and middle-aged adults.

### Reduced activation-induced expansion of CMV-specific CD8^+^ cells in vitro

**Fig. 2:**
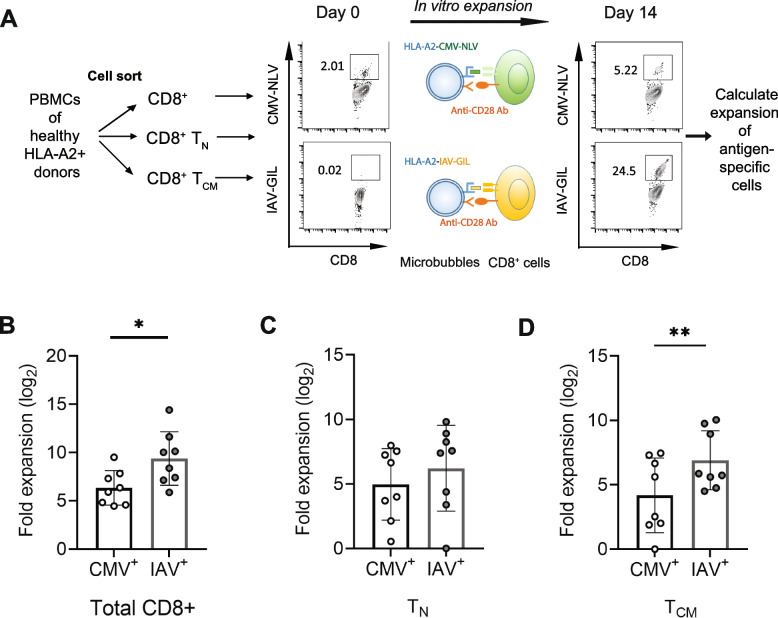
Reduced *in vitro* expansion of CMV-specific CD8^+^ T_CM_ cells compared to IAV-specific CD8^+^ T_CM_ cells. **A** Experimental design for the expansion of CMV- and IAV-specific CD8^+^ T cells by artificial antigen presenting cell (aAPC) *in vitro*. **B**-**D** Expansion of CMV- and IAV- specific CD8^+^ T cells (Total, T_N,_ and T_CM_) from the same CMV IgG positive healthy adults (*N*=10). * Indicates *P* < 0.05, ** indicates *P* < 0.01

To determine the functional changes of CMV-specific CD8^+^ T cells, we isolated CD8^+^ T cells from CMV IgG positive subjects and cultured them in the presence of the CMV epitope or IAV epitope using the artificial APC culture system [31]. After 14 days of culture, CMV- and IAV-specific CD8^+^ T cells were analyzed by dextramer or tetramer staining and flow cytometry, and the expansion of antigen-specific CD8^+^ T cells was calculated based on the seeded and harvested antigen-specific cells (Fig. 2A). Compared to that of IAV-specific cells, the expansion of CMV-specific cells was significantly reduced (4.0 ± 1.7-fold) (Fig. 2B). To further determine which types of CD8^+^ T cells contributed to this reduced expansion, we isolated T_N_ and T_CM_ of CD8^+^ T cells by cell sorting and cultured using the artificial APC system. We found that there was a similar expansion between CMV- and IAV-specific T_N_ cells (Fig. 2C) but significantly lower (2.4 ± 0.6-fold) expansion of CMV-specific T_CM_ than that of IAV-specific T_CM_ cells (Fig. 2D). These findings revealed that there was no intrinsic difference in response to *in vitro* stimulation between CMV- and IAV-specific CD8^+^ T_N_ cells and CMV infection reduced robustness of activation-induced expansion of CMV-specific T_CM_ CD8^+^ cells in healthy young and middle-aged adults.

### Reduced CD70 expression and expansion of CMV-specific T_CM_ cells

**Fig. 3:**
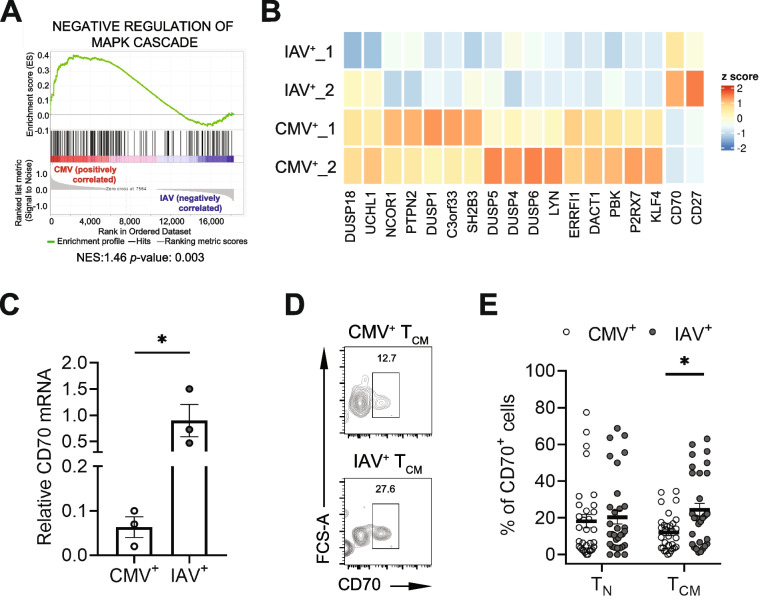
Reduced expression of CD70 in CMV-specific CD8^+^ T_CM_ cells. **A** Gene Set Enrichment Analysis (GSEA) identified genes in pathway negatively regulating MAPK activity which is enriched in CMV-specific T_CM_ cells. **B** Heatmap showing genes in pathway negatively regulating MAPK activity differentially express between CMV-specific and IAV-specific T_CM_ cells from duplicated microarray experiments. **C** The expression level of CD70 in CMV- and IAV-specific T_CM_ cells analyzed by RT-PCR. **D** Representative flow cytometry plots showing gating on CD70 expression in CMV- and IAV-specific CD8^+^ T cells. (**E**) Frequencies of CD70^+^ cells in CMV- and IAV-specific T_N_ (*N*=32) and T_CM_ (*N*=33) cells from CMV IgG positive healthy adults. * Indicates *P* < 0.05

To determine the transcriptome changes that contribute to reduced proliferation of CMV-specific T_CM_ CD8^+^ cells, we compared global gene expression between CMV- and IAV-specific CD8^+^ T_CM_ cells using microarray technology and analyzed the functional changes based on highly expressed genes between CMV- and IAV-specific CD8^+^ T_CM_ cells by gene set enrichment analysis (GSEA). As shown in Supplemental Table S2, 6 gene sets are significant at p<0.05. Negative regulation of the MAPK pathway was enriched in CMV-specific T_CM_ CD8^+^ cells (Fig. 3A). Given the involvement of MAPK activation in CD70 signaling in T and B cells [32, 33], it is noteworthy that CD70 is one of the genes whose mRNA levels were decreased in CMV-specific compared with IAV-specific CD8^+^ T_CM_ cells (Fig. 3B). A reduced level of mRNA of CD70 in CMV-specific CD8^+^ T cells was confirmed by RT-PCR (Fig. 3C). Furthermore, the percentage of CD70 expressing cells was reduced in CMV-specific compared to IAV-specific CD8^+^ T_CM_ cells (15.5% ± 2.6% vs. 25.0% ± 3.3%, p=0.012) by flow cytometry analysis (Fig. 3D-E). Collectively, we observed a significantly reduced number of CD70 expressing CMV-specific T_CM_ cells but not T_N_ cells compared to the IAV-specific counterparts (Fig. 3E).

**Fig. 4:**
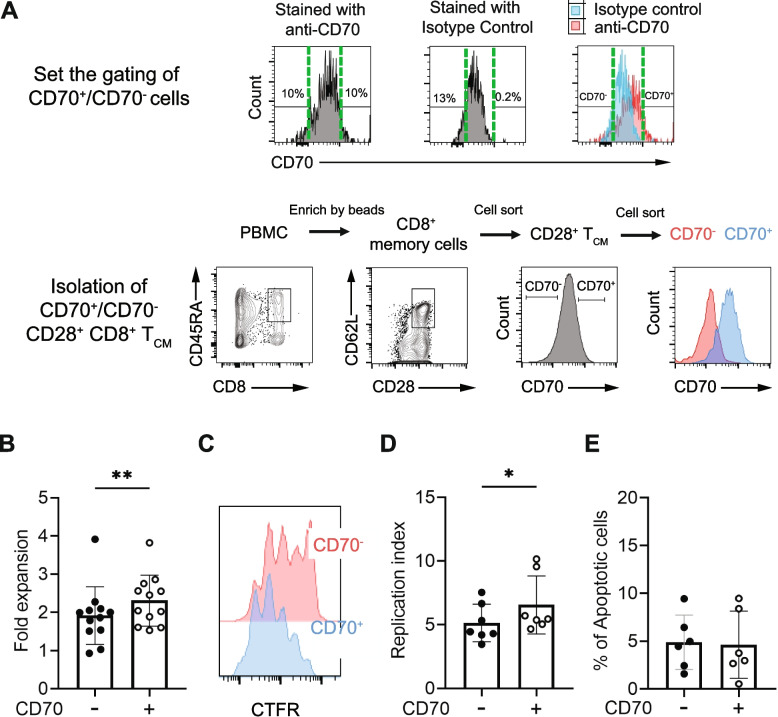
Correlation of CD70 expression and cell division/expansion of CD8^+^ T_CM_ cells *in vitro*. **A** Gating strategy for CD70^+^ and CD70^-^ cells. Representative flow cytometry plots showing isolating CD70^+^ and CD70^-^ cells from CD28^+^ CD8^+^ T_CM_ cells using FACS sort. **B** CD70^+^ cells expanded better than CD70^-^ cells *in vitro*. Sorted CD70^+^ and CD70^-^ CD8^+^ T_CM_ cells were stimulated with anti-CD3/CD28 for 4 days (*N*=12). **C** Representative flow cytometry plots of cell division. Sorted CD70^+^ and CD70^-^ CD8^+^ T_CM_ cells were labeled with CTFR and stimulated with anti-CD3/CD28 for 4 days. Cell divisions were analyzed by Modfit, and replication index was presented (*N*=6). **D** CD70^+^ CD8^+^ T_CM_ cells had more cell divisions than CD70^-^ CD8^+^ T_CM_ cells as measured by the replication index (*N*=6). **E** Percentage of apoptotic cells in CD70^+^ and CD70^-^ CD8^+^ T_CM_ cells after 4 days of anti-CD3/CD28 stimulation (*N*=6). * Indicates *P* < 0.05, ** indicates *P* < 0.01

It has been reported that helper B cells promote CD8^+^ T cell proliferation via the CD70 signaling pathway [28]. It is of interest to determine if the level of CD70 expression in CD8^+^ T cells regulates their activation-induced expansion. To address this, we sorted CD28^+^ T_CM_ cells into CD70^+^, defined as the highest 10% CD70 expression, and CD70^-^, defined as the lowest 10% CD70 expression, based on isotype control staining (Fig. 4A) and stimulated with anti-CD3/CD28. We found that CD70^+^ T_CM_ cells expanded modestly but significantly better than did CD70^-^ T_CM_ cells *in vitro* (Fig. 4B). To determine if the better expansion of CD70^+^ T_CM_ cells was due to increased cell division or reduced cell death, we analyzed cell divisions of CD70^+^ and CD70^-^ T_CM_ by cell trace far red (CTFR) and Annexin V. We found that CD70^+^ T_CM_ cells had significantly more cell division than CD70^-^ T_CM_ cells (measured by the replication index, 6.5 ± 0.9 vs. 5.1 ± 0.6) (Fig. 4C-D). There were no substantial differences in the percentage of apoptotic cells between CD70^+^ and CD70^-^ T_CM_ cells (Fig. 4E). These findings showed that the CD70 expression correlates with better activation-induced CD8^+^ T_CM_ cell division *in vitro*.

### CD70 signaling via ERK1/2 phosphorylation and reduced in CMV-specific CD8^+^ T_CM_ cells

**Fig. 5:**
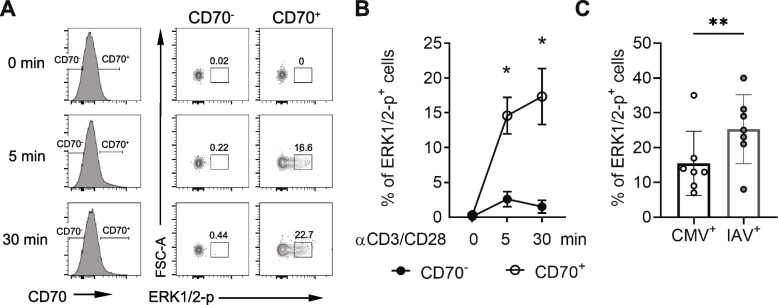
CD70 expression correlated with activation-induced phosphorylation of ERK1/2. **A** A representative flow cytometry plot of ERK1/2 phosphorylation in CD70^+^ and CD70^-^ CD8^+^ memory T cells before and after anti-CD3/CD28 stimulation *in vitro* at 5 and 30 minutes. Isolated CD8^+^ T memory cells were treated with anti-CD3/CD28 for indicated time and cells were then fixed and stained for CD70 and phosphorylated ERK1/2. **B** Percentage of phosphorylated ERK1/2 positive in CD70^+^ and CD70^-^ CD8^+^ T memory cells. The data were the average of four independent experiments. **C** ERK1/2 phosphorylation in CMV- and IAV-specific CD8^+^ T_CM_ cells after anti-CD3/CD28. 1 x 10^4^ sorted CMV- and IAV-specific CD8^+^ T_CM_ cells (N=7) were stimulated with anti-CD3/CD28 for 15 min, fixed with PFA, permeabilized with cold methanol, and stained with antibody against phosphorylated ERK1/2 (2 x 10^5^ 293T cells were added to reduced cell loss during centrifugation). Percentages of phosphorylated ERK1/2 positive antigen-specific CD8^+^ T_CM_ cells were back-calculated based on the ratio between antigen-specific CD8^+^ T_CM_ cells and 293T cells. * Indicates *P* < 0.05, ** indicates *P* < 0.01

**Fig. 6:**
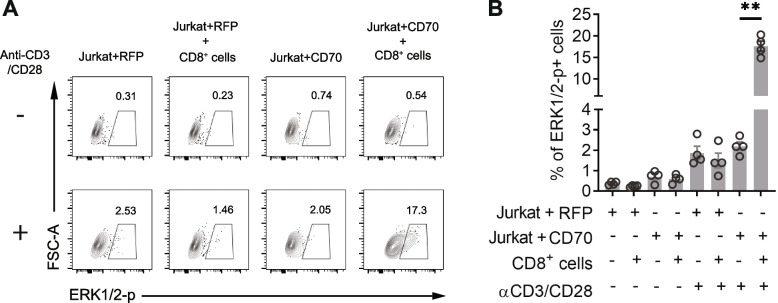
CD70 signaling through phosphorylation of ERK1/2 in T cells (**A**) Jurkat cells expressing CD70 or mRFP control were mixed (1:1) with CD8^+^ T cells and stimulated with anti-CD3/CD28. ERK1/2 phosphorylation in Jurkat cells was analyzed by flow cytometry. Representative flow cytometry plots were shown. **B** Quantification of four independent experiments as described in A. ** indicates *P* < 0.01

MAPK activation is involved in CD70 signaling [32, 33], To determine if CD70^+^ T_CM_ cells receive the signal via interaction with CD27, we first compared the activity of the MAPK pathway by determining the level of ERK1/2 phosphorylation in CD70^+^ and CD70^-^ memory CD8^+^ T cells after anti-CD3/CD28 stimulation and found a more rapidly increased ERK1/2-phosphorylation after *in vitro* stimulation in CD70^+^ than in CD70^-^ memory CD8^+^ T cells (Fig. 5A-B). We then examined whether the activity of the MAPK pathway was reduced in CMV-specific T_CM_ cells by directly comparing ERK1/2 phosphorylation of sorted CMV- and IAV-specific T_CM_ cells post anti-CD3/CD28 stimulation. Indeed, we found that ERK1/2 phosphorylation was significantly decreased in CMV-specific T_CM_ cells compared to the IAV-specific T_CM_ cells of the same subject (15.4% ± 3.5% vs. 25.3% ± 3.7%, p=0.0079) (Fig. 5C and Supplemental Fig. S1B-C). This was consistent with the observation that CMV-specific T_CM_ cells had fewer CD70^+^ cells compared to IAV-specific T_CM_ cells (Fig. 3). However, the difference of CD70^+^ T_CM_ cells might not be the only difference between gated CD70^+^ and CD70^-^ cells that could potentially enhance ERK1/2-phosphorylation. To directly demonstrate that the CD70 signaling pathway resulted in increased ERK1/2 phosphorylation after engaging CD27, we expressed CD70 in Jurkat cells, a human T cell line that does not express either CD70 or CD27 (Supplemental Fig. S2). Expression of CD70 in Jurkat cells was not sufficient to induce ERK1/2 phosphorylation even after anti-CD3/CD28 stimulation on their own. However, when we added CD8^+^ T cells that express CD27 (Supplemental Fig. S2), cells expressing ERK1/2 phosphorylation increased over 30-fold. Importantly, Jurkat cells that lack CD70 expression were unable to induce ERK1/2 phosphorylation after anti-CD3/CD28 stimulation even in the presence of CD27-expressing CD8^+^ T cells (Fig. 6A-B). Together, these findings revealed that engaging CD70 signals through the MAPK pathway during T cell activation and reduced CD70 expression in CMV-specific T_CM_ cells causes dampened MAPK activity and reduced expansion *in vitro*.

### Stable difference of reduced CD70 expression between CMV- and IAV-specific T_CM_ cells with age

**Fig. 7:**
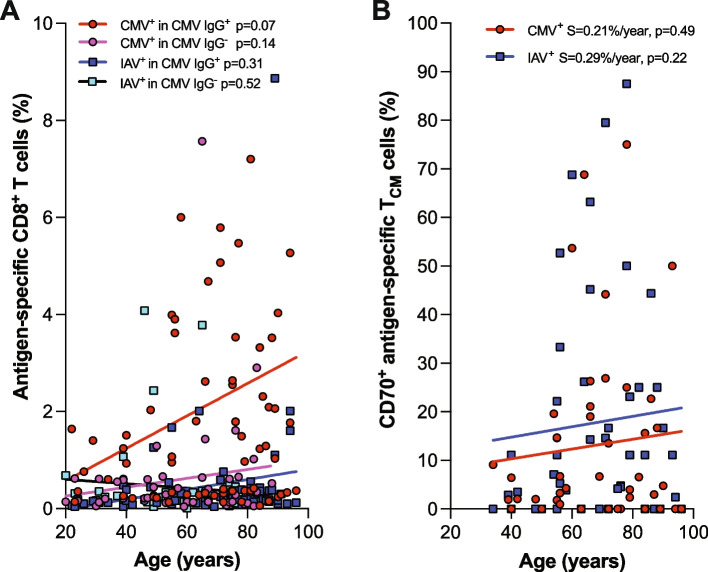
Stable reduced expression of CD70 in CMV-specific T_CM_ compared to IAV-specific T_CM_ with age. **A** Age-related changes in CMV- and IAV-specific CD8^+^ T cells in CMV IgG^+^ and IgG^-^ donors. The *p*-value for correlation between CMV-specific cells and age is 0.066. The difference between the age-adjusted average percentage of CMV-specific and IAV-specific cells in IgG^+^ donors is significant (*p*=0.0005). Similarly, the difference of age-adjusted average percentage of CMV-specific cells between IgG^+^ and IgG^-^ donors is significant (*p*=0.0012). **B** CD70 expression in CMV- and IAV-specific T_CM_ in CMV IgG^+^ subjects. A cohort of 45 healthy CMV IgG^+^ subjects aged from the 20s to 80s was analyzed via tetramer staining by flow cytometry

Expansion of CMV-specific CD8^+^ T cells, particularly differentiated CD8^+^ memory cells, was observed in young and middle-aged healthy adults (Fig. 1). We confirmed that CMV-specific CD8^+^ T cells were further increased with aging in CMV IgG^+^ donors (Fig. 7A). As CD70^+^CD8^+^ T cells also increase with age [30], we wanted to determine if the age-related increase of CD70^+^CD8^+^ T cells alters the reduced CD70^+^ CMV-specific T_CM_ cells compared to the IAV-specific T_CM_ cells. We compared CD70^+^ CMV- and IAV-specific T_CM_ cells of the same subjects and found the difference of CD70^+^ CMV- and IAV-specific T_CM_ cells was stable in our study cohort (aged from the 20s to 80s) (Fig. 7B). This showed that the age-related increase of CD70 expression in CD8^+^ T cells in both CMV- and IAV- specific T_CM_ cells and that aging does not alter the difference of higher number of CD70^+^ IAV-specific T_CM_ cells than that of CD70^+^ CMV- T_CM_ cells.

## Discussion

The history of CMV and CD8^+^ T cell interactions in a host has been proposed into three stages: the initial acute infection, the long controlled latent reactivation, and the eventual memory inflation or senescence [13]. Inflation of CMV-specific memory CD8^+^ T cells are considered one of the hallmarks of impaired immune function in the old population but the impact of CMV infection on CD8^+^ T cell function during the second stage of this interaction in healthy young and middle-aged adults has not been fully examined. Here, we show that CMV infection causes mild expansion and differentiation of CMV-specific CD8^+^ T cells compared to IAV-specific CD8^+^ T cells in young and middle-aged healthy adults. This manifests not only an increased number of three memory subsets (T_SCM_, T_EM,_ and T_EMRA_) of CMV-specific CD8^+^ cells but also altered expression of several key proteins: reduced expression of IL7R and two key co-stimulatory molecules (CD28 and CD27) in memory subsets (T_SCM_, T_CM,_ and T_EMRA_). Noticeably, the expression of granzyme B and perforin was not changed, suggesting that the differentiation of CMV-specific memory CD8^+^ cells in healthy adults are not in a terminally differentiated status, which is associated T cell aging. Furthermore, the IAV-specific memory CD8^+^ cell subsets have no obvious changes between CMV IgG positive and negative healthy subjects, indicating that CMV-infection induced alteration is antigen-specific in these healthy subjects. Collectively, our findings indicate that chronic infection of CMV causes expansion and differentiation of only CMV-specific memory CD8^+^ cells but not IAV-specific memory CD8^+^ cells of the same subjects.

Activation-induced proliferation and expansion is a key functional feature of CD8^+^ T cells. Here we compared the *in vitro* expansion of CMV- and IAV-specific CD8^+^ T cells and demonstrated a significantly reduced expansion of CMV-specific CD8^+^ T cells compared to the IAV-specific CD8^+^ T cells. This difference is not found in antigen-specific T_N_ cells but rather in antigen-specific T_CM_ cells. This suggests that there is no intrinsic difference of CD8^+^ T_N_ cells to these different viral epitopes and that reduced expansion of CMV-specific T_CM_ cells is likely due to repeated stimulation of latent infection of CMV. This conclusion is supported by the evidence that CMV-specific memory CD8^+^ T cells are expanded *in vivo* and have a more differentiated status. Collectively, these findings suggest alteration of CMV-specific CD8^+^ T cells is a gradual process during CMV infection.

CD27-CD70 co-stimulation provides a critical signaling pathway of CD8^+^ T cell immunity [21]. Binding of CD70 to CD27 on T cells leads to recruitment of TNFR-associated factor (TRAF) proteins to the CD27 cytoplasmic tail [34, 35], which in turn activate canonical and non-canonical nuclear factor-kB (NF-kB) and c-Jun-N-terminal kinase (JNK) signaling pathways to elicit T cell responses. Interestingly, reverse signaling via interaction of CD27 to CD70 on B cells [32], mouse CD8 T cells [29], and on NK cells [36] activates MARK and PI3K signaling pathways. However, it has not been determined whether the same CD70 signaling pathway functions in human memory T cells. Our findings here demonstrate that CD70 receives a signal from CD27 resulting in phosphorylation of ERK1/2. This signaling was reduced in CMV-specific T_CM_ cells due to reduced CD70 expression, providing a partial explanation for the reduced expansion of CMV-specific T_CM_ CD8^+^ T cells *in vitro*. This is reminiscent of the finding that blocking CD70 interaction with CD27 reduced EBV specific CD8 T cell proliferation [37] and CD70 expression on CD8^+^ T cells enhances proliferation when B cells provide a helper function via CD27 [28]. Compared to the well-studied role of CD27 in T cell activation and maintenance, the signaling through CD70 is less studied in T cells. Whether CD70 signaling provides a function in addition to proliferation in human memory T cells remains to be determined. Potential sources of CD27 that can engage CD70 expressing CD8^+^ T cells *in vivo* include autologous interaction with neighboring CD8^+^ T cells, CD4^+^ T cells, and B cells. It remains to be determined whether CD70-CD27 interaction between CD8^+^ T cells and CD4^+^ T cells plays a role in the development and maintenance of CTL response.

## Conclusion

Chronic CMV infection is associated with age associated alteration of CD8^+^ T cell functions. While CD70^+^CD8^+^ T cells including CD70^+^ CD8^+^ T_CM_ increase with age, reduced CD70^+^ CMV-specific T_CM_ remained relatively stable throughout five decades of adult life. Thus, the impact of persistent infection on CMV-specific memory CD8^+^ T cells is consistent with changes seen in aging. In conclusion, our study reveals the alterations of CMV-specific memory CD8^+^ T cells associated with chronic CMV infection in healthy young and middle-aged adults. It is of great interest to determine whether decreased CD70 expression is a common sign for reduced memory CD8^+^ T cell function in other chronic viral infections and whether modulating CD70 expression offers a potential means to enhance CD8^+^ T cell proliferation against infections and cancers.

## Methods

### Selection of study participants and isolation of CD8^+^ T cells from blood of healthy adult humans

Healthy adult humans were recruited from the NIA clinic and NIH blood bank under the NIH IRB-approved protocols*.* Study participants were selected based on the following criteria: HLA-A2^+^ and aged 18 and older. Blood was collected from these selected donors via apheresis or regular blood donations. Peripheral mononuclear blood cells (PBMCs) were isolated from blood by Ficoll-gradient centrifugation. CD8^+^ T cells were enriched from PBMCs by negative immune selection with antibodies cocktail and BigMag goat anti-mouse IgG beads (Qiagen) as described previously [38]. The purity of CD8^+^ T cells was 80%-90%. CD8^+^ T_N_ and T_CM_ cells were further isolated by cell sort (MoFlo, Beckman Coulter) based on the expression of CD8, CD45RA, and CD62L (Biolegend) and their purities were over 90%. The source and titration of antibodies used in flow cytometry analysis of this study was summarized in the Supplementary Table S2.

### Expansion of antigen-specific CD8^+^ T cell and subsets using the artificial antigen presentation cell system (aAPC)

Antigen-specific CD8^+^ T cells were expanded using the artificial antigen presentation system in vitro [31]. In short, freshly isolated CD8^+^ T cells by immunoseparation procedure or CD8^+^ T_N_ and T_CM_ cells by cell sort and were counted, and stimulated with peptide-loaded (either CMV-pp65_495-503_, NLVPMVATV or IAV-M1_58-66_, GILGFVFTL) (Peptide2go) aAPC beads containing HLA-A2-Ig plus anti-CD28 conjugated Dynal beads [31] or lipid microbubble [39] with peptide final concentration of 10^-8^ M in 96-well round bottom plates (a total of 2 x 10^6^ CD8^+^ T cells per antigen) for 14 days. Each well contained 1 x 10^4^ CD8^+^ T cells and 1x10^4^ peptide-loaded aAPC beads in 160 μl M1 medium. M1 medium was RPMI-1640 containing 5% human autologous sera, 0.11 nM β-mercaptoethanol (Gibco 21985-023), 1 mM Sodium Pyruvate (Sigma S8636), 0.1 mM non-essential amino acids in MEM (Gibco 11140-050), 1X MEM-Vitamins (Gibco 11120-52) and 1X PenStrep (Gibco 15140-122). Four days into stimulation, 80 μl of M2 media (10% human autologous sera and 8% TFs in M1 media) was added to each well and cultured for another 2 days. On day 7, cells were harvested, counted, and CD8^+^ T cells were stained with antigen-specific dextramers. Expanded cells were seeded at 1 x 10^4^ CD8^+^ T cells/well for the second round of aAPC culture, using an equal number of aAPC beads, for another seven days. On day 14, cells were counted and stained with their respective tetramers and the antigen-specific cells were calculated. The *in vitro* expansion was calculated by the number antigen-specific CD8^+^ T cells at day 14 divided by day 0.

### Phenotypic analysis of CMV- and IAV-specific CD8^+^ T cells by flow cytometry

The procedure of flow cytometric analysis of CMV- and IAV-specific CD8^+^ T cells prior to and post *in vitro* stimulation was described [40]. Briefly, freshly PMBCs were stained with fluorescently labeled dextramers or tetramers specific to CMV-pp65 (NLVPMVATV) and IAV-M1 (GILGFVFTL) (Immudex, Copenhagen, Denmark and NIAID tetramer core) first at room temperature for 20 min; followed by staining of cell surface markers including antibody against CD3, CD8, CD62L, CD45RA, CD95, CD27, CD28 CD70, CD127, and CD69 at 4°C for 30 min. Cells were washed again with FACS buffer (Hanks solution with 0.3% Sodium Azide), and then fixed immediately with 3% formaldehyde and 1% FBS in FACS buffer. Fixed cells were further stained with intracellular markers (perforin and granzyme B) at 4°C for 30 min. Stained cells were collected by BD_Symphony and were analyzed using FlowJo version 7.6.5 software.

Antibodies (CD3-BV570, CD62L-FITC, CD62L-PE-Cy7, CD45RA-PE-Cy7, CD45RA-APC, CD95-PE-Cy5, CD27-PE, CD28-BV785, CD28-PerCP-Cy5.5, CD70-PE, CD127--BV711, CD69-BV650, and granzyme B) were purchased from Biolegend, and antibodies against CD8-BUV496, CD27-BUV395, and perforin-PE-CF594 were purchased from BD.

### Microarray for gene expression analysis

The procedure of microarray analysis was previously described [33]. Briefly, total RNA was extracted from freshly isolated CMV- and IAV-specific CD8^+^ T cells using the RNeasy Mini kit (Qiagen). RNA quantity and quality were measured using a NanoDrop 2000 and a 2100 Bioanalyser and 400 ng of total RNA was used for cRNA synthesis using LowInput QuickAmp labeling kit (Agilent). Four pooled RNA samples were used to make Cy3-labelled cRNA and Cy5-labelled universal human reference RNA (Agilent). 750 ng of labeled sample probes mixed with an equal amount of universal reference probes in 500 μl of hybridization solution onto Agilent SurePrint G3 Human Gene Expression 8x60 K microarrays chip (Agilent) at 60 °C for 17 hours according to manufacturer's instructions. Following hybridization for 40 hours, microarray slides were washed and then scanned. The output file consisted of processed signal intensities from Cy3 and Cy5 fluorescent channels using Feature Extraction software (Agilent). Two independent microarray experiments were performed for CMV- and IAV-specific CD8^+^ T cells. A modified ANOVA analysis was used on log-transformed data and the statistical significance was determined using the false discovery rate (FDR) using a web-based analysis software (NIA array analysis, http://lgsun.grc.nia.nih.gov/ANOVA/). Gene Set Enrichment Analysis (GSEA) was performed using GSEA v2.2.4 (Broad Institute, Cambridge, USA) to determine which gene sets are enriched in expression in CMV-specific T_CM_ compared to IAV-specific T_CM_ cells. The thresholds for the nominal p-value were set to <0.05. The data were deposited to NCBI (GSE200258).

### Isolation and stimulation of CD70^+^ and CD70^-^ CD8^+^ T_CM_ cells in vitro

Memory CD8^+^ T cells were isolated by immunomagnetic isolation and followed by cell sort. Cells were stained with CD8, CD45RA, CD62L, CD27, CD28, and CD70 (Biolegend) and gated on CD8^+^CD45RA^-^CD62L^+^CD28^+^CD27^+^ and sorted for CD70^+^ and CD70^-^T_CM_ cells by MoFlo XDP (Beckman Coulter). We used PBMC of a healthy adult for CD70 staining standard and the highest and lowest 10% of CD70 expressed T_CM_ cells were defined as CD70^+^ and CD70^-^ cells and this definition was applied to all study subjects of every sort (Fig. 4A). Sorted CD70^+^ and CD70^-^ CD8^+^ T_CM_ cells were stimulated by anti-CD3/CD28 antibodies for 4 days and cells were harvested for cell count, apoptosis analysis using AnnexinV/7-AAD Apoptosis Detection Kit (Biolegend) according to the manufacturer’s instructions. For cell division assay, sorted CD70^+^ and CD70^-^ cells were washed with PBS and labeled with CellTrace Far Red (CTFR, Thermo Fisher Scientific) according to the manufacturer’s instructions. Dilution of CTFR was analyzed by flow cytometry after cells were stimulated with anti-CD3/CD28 antibodies for 4 days and replication index (the number of daughter cells divided by the number of cells in the original culture that divided) was calculated by using Modfit software (Verity Software House).

### CD70 expression in Jurkat cells

CD70 gene (NM_001252) was amplified from cDNA of CD8^+^ memory T cells using primers 5’-gtacgcggccgcATGCCGGAGGAGGGTTCGGGC-3’ and 5’-gtacgctagcTCAGGGGCGCACCCACTGCACTC-3’) and cloned into pHAGE-mRFP1 (gift from Dr. Xin Lin, Tsing Hua University, China) between *NotI* and *NheI* sites. Lentivirus were generated via transfection of 293T cells. Briefly, 8 × 10^6^ 293T cells were seeded in a 100 mm plate to reach 80% of confluence on the day of virus packing. The 2nd generation lentiviral packaging plasmids CMV-dR8.2 and pCMV-VSV-G (Addgene) were co-transfected with pHAGE-mRFP1 empty vector or pHAGE-mRFP1-CD70 using FuGENE HD (Promega). The supernatant containing virus was collected 2 and 3 days after transfection and concentrated using Lenti-X Concentrator (Takara Bio). Concentrated viral supernatant was transduced in Jurkat cells using spinoculation with 5 μg/ml of polybrene (Sigma-Aldrich). Stably transduced cells were then selected based on the expression of mRFP and cell sorting after 4 days of transduction.

### Measurement of ERK1/2 phosphorylation

Cells were stimulated with anti-CD3/CD28 for indicated time and fixed with 2% paraformaldehyde (PFA) at 4 °C for 10 min. Fixed cells were resuspended in cold methanol at 4 °C for 30 min. When tested CD8^+^ T cells were fewer than 1x10^4^, 2x10^5^ 293T cells were added to permeabilized cells in methanol before centrifugation to help the recovery of cells during subsequent washing steps. Cells were washed twice with 1x Perm/Wash buffer (BD Biosciences). Cells were resuspended in 1x Perm/Wash buffer and stained with anti-ERK1/2 Phospho (Thr202/Tyr204) antibody (Biolegend) at 4 °C for 30 min. Samples were washed with 1x Perm/Wash buffer and resuspended in 1% PFA in PBS. Data were acquired on a BD Canto II flow cytometer (BD Biosciences) and results were analyzed with FlowJo.

### Measurement of CMV IgG in blood

Measurement of blood CMV IgG was previously described [41]. Briefly, 50 μl of plasma was diluted in PBS and used for the ELISA kit (Abcam, # ab108639) according to the manufacturer’s instructions. Antibody titers were calculated using the kit standard. Values below 1 were considered negative and above 1 was considered as positive.

### Statistical analysis

Correlation test and Standard two-tailed Student T-test were used for analysis. Asterisk on graphs represent *=*p*<0.05, **=*p*<0.01, and **=*p*<0.001. Group differences between CMV IgG+ and IgG- subjects of T cells were compared using a separate linear regression model with each T cell as the outcome. The main predictor of the model was group, with covariates of age and sex. We used the type 1 error of 0.05, selecting comparisons with a p-value less than that as significant. All analyses were performed using R version 4.1.0.

## Supplementary Information

**Additional file 1: Supplementary Figure 1. **Expression of different markers inantigen-specific CD8^+^ cells. **Supplementary Figure 2. **Percentageof CD28^+^ antigen-specific T_N_, T_CM_ and totalCD8^+^ Tcells. **Supplementary Figure 3.** ERK1/2 phosphorylation in sorted IAV^+^and CMV^+^ T_CM_ cells. **Supplementary Figure 4.** CD70 expression in Jurkat cells and CD27-CD70 signaling.

**Additional file 2: Supplementary Table 1. **Demographics of study subjects used in the figures(experiments). **Supplementary Table 2.** GO pathways identified by GSEA analysis. **Supplementary Table 3.** Antibody list and information used in flowcytometry analysis.

## Data Availability

Microarray data were deposited to NCBI (GSE200258).

## References

[1] Tscharke DC, Croft NP, Doherty PC, La Gruta NL (2015). Sizing up the key determinants of the CD8(+) T cell response. Nat Rev Immunol..

[2] Reina-Campos M, Scharping NE, Goldrath AW (2021). CD8(+) T cell metabolism in infection and cancer. Nat Rev Immunol..

[3] Akondy RS, Fitch M, Edupuganti S, Yang S, Kissick HT, Li KW (2017). Origin and differentiation of human memory CD8 T cells after vaccination. Nature..

[4] Grant EJ, Quinones-Parra SM, Clemens EB, Kedzierska K (2016). Human influenza viruses and CD8(+) T cell responses. Curr Opin Virol..

[5] Zuniga EI, Macal M, Lewis GM, Harker JA (2015). Innate and adaptive immune regulation during chronic viral infections. Annu Rev Virol..

[6] Pawelec G (2014). Immunosenenescence: role of cytomegalovirus. Exp Gerontol..

[7] Sansoni P, Vescovini R, Fagnoni FF, Akbar A, Arens R, Chiu YL (2014). New advances in CMV and immunosenescence. Exp Gerontol..

[8] Nikolich-Zugich J, Cicin-Sain L, Collins-McMillen D, Jackson S, Oxenius A, Sinclair J (2020). Advances in cytomegalovirus (CMV) biology and its relationship to health, diseases, and aging. Geroscience..

[9] Hassouneh F, Goldeck D, Pera A, van Heemst D, Slagboom PE, Pawelec G (2021). Functional changes of T-cell subsets with age and CMV infection. Int J Mol Sci..

[10] Dioverti MV, Razonable RR (2016). Cytomegalovirus. Microbiol Spectr..

[11] Staras SA, Dollard SC, Radford KW, Flanders WD, Pass RF, Cannon MJ (2006). Seroprevalence of cytomegalovirus infection in the United States, 1988-1994. Clin Infect Dis..

[12] Miles DJ, Sanneh M, Holder B, Crozier S, Nyamweya S, Touray ES (2008). Cytomegalovirus infection induces T-cell differentiation without impairing antigen-specific responses in Gambian infants. Immunology..

[13] Klenerman P, Oxenius A (2016). T cell responses to cytomegalovirus. Nat Rev Immunol..

[14] Dunn W, Chou C, Li H, Hai R, Patterson D, Stolc V (2003). Functional profiling of a human cytomegalovirus genome. Proc Natl Acad Sci U S A..

[15] Cunningham C, Gatherer D, Hilfrich B, Baluchova K, Dargan DJ, Thomson M (2010). Sequences of complete human cytomegalovirus genomes from infected cell cultures and clinical specimens. J Gen Virol..

[16] Reus B, Caserta S, Larsen M, Morrow G, Bano A, Hallensleben M (2021). In-depth profiling of T-cell responsiveness to commonly recognized CMV antigens in older people reveals important sex differences. Front Immunol..

[17] Sylwester A, Nambiar KZ, Caserta S, Klenerman P, Picker LJ, Kern F (2016). A new perspective of the structural complexity of HCMV-specific T-cell responses. Mech Ageing Dev..

[18] Sylwester AW, Mitchell BL, Edgar JB, Taormina C, Pelte C, Ruchti F (2005). Broadly targeted human cytomegalovirus-specific CD4+ and CD8+ T cells dominate the memory compartments of exposed subjects. J Exp Med..

[19] Vescovini R, Fagnoni FF, Telera AR, Bucci L, Pedrazzoni M, Magalini F (2014). Naive and memory CD8 T cell pool homeostasis in advanced aging: impact of age and of antigen-specific responses to cytomegalovirus. Age.

[20] Acuto O, Michel F (2003). CD28-mediated co-stimulation: a quantitative support for TCR signalling. Nat Rev Immunol..

[21] Grant EJ, Nussing S, Sant S, Clemens EB, Kedzierska K (2017). The role of CD27 in anti-viral T-cell immunity. Curr Opin Virol..

[22] Ueda Y, Levine BL, Huang ML, Freeman GJ, Nadler LM, June CH (1995). Both CD28 ligands CD80 (B7-1) and CD86 (B7-2) activate phosphatidylinositol 3-kinase, and wortmannin reveals heterogeneity in the regulation of T cell IL-2 secretion. Int Immunol..

[23] Weng NP, Akbar AN, Goronzy J (2009). CD28(-) T cells: their role in the age-associated decline of immune function. Trends Immunol..

[24] Riddell NE, Griffiths SJ, Rivino L, King DC, Teo GH, Henson SM, et al. Multifunctional cytomegalovirus (CMV)-specific CD8 T cells are not restricted by telomere-related senescence in young or old adults. Immunology. 2014. 10.1111/imm.12409 PMC4368162 25314332

[25] Denoeud J, Moser M (2011). Role of CD27/CD70 pathway of activation in immunity and tolerance. J Leukocyte Biol..

[26] van Gisbergen KP, Klarenbeek PL, Kragten NA, Unger PP, Nieuwenhuis MB, Wensveen FM (2011). The costimulatory molecule CD27 maintains clonally diverse CD8(+) T cell responses of low antigen affinity to protect against viral variants. Immunity..

[27] Welten SP, Redeker A, Franken KL, Benedict CA, Yagita H, Wensveen FM (2013). CD27-CD70 costimulation controls T cell immunity during acute and persistent cytomegalovirus infection. J Virol..

[28] Deola S, Panelli MC, Maric D, Selleri S, Dmitrieva NI, Voss CY (2008). Helper B cells promote cytotoxic T cell survival and proliferation independently of antigen presentation through CD27/CD70 interactions. J Immunol..

[29] Arens R, Schepers K, Nolte MA, van Oosterwijk MF, van Lier RA, Schumacher TN (2004). Tumor rejection induced by CD70-mediated quantitative and qualitative effects on effector CD8+ T cell formation. J Exp Med..

[30] Wang D, Du J, Song Y, Wang B, Song R, Hao Y (2020). CD70 contributes to age-associated T cell defects and overwhelming inflammatory responses. Aging.

[31] Oelke M, Maus MV, Didiano D, June CH, Mackensen A, Schneck JP (2003). Ex vivo induction and expansion of antigen-specific cytotoxic T cells by HLA-Ig-coated artificial antigen-presenting cells. Nat Med..

[32] Arens R, Nolte MA, Tesselaar K, Heemskerk B, Reedquist KA, van Lier RA (2004). Signaling through CD70 regulates B cell activation and IgG production. J Immunol..

[33] Taraban VY, Rowley TF, Al-Shamkhani A (2004). Cutting edge: a critical role for CD70 in CD8 T cell priming by CD40-licensed APCs. J Immunol..

[34] Akiba H, Nakano H, Nishinaka S, Shindo M, Kobata T, Atsuta M (1998). CD27, a member of the tumor necrosis factor receptor superfamily, activates NF-kappaB and stress-activated protein kinase/c-Jun N-terminal kinase via TRAF2, TRAF5, and NF-kappaB-inducing kinase. J Biol Chem..

[35] Gravestein LA, Amsen D, Boes M, Calvo CR, Kruisbeek AM, Borst J (1998). The TNF receptor family member CD27 signals to Jun N-terminal kinase via Traf-2. Eur J Immunol..

[36] Al Sayed MF, Ruckstuhl CA, Hilmenyuk T, Claus C, Bourquin JP, Bornhauser BC (2017). CD70 reverse signaling enhances NK cell function and immunosurveillance in CD27-expressing B-cell malignancies. Blood..

[37] Deng Y, Chatterjee B, Zens K, Zdimerova H, Muller A, Schuhmachers P (2021). CD27 is required for protective lytic EBV antigen-specific CD8+ T-cell expansion. Blood..

[38] Araki Y, Wang Z, Zang C, Wood WH, Schones D, Cui K (2009). Genome-wide analysis of histone methylation reveals chromatin state-based regulation of gene transcription and function of memory CD8+ T cells. Immunity..

[39] Lustig A, Manor T, Shi G, Li J, Wang YT, An Y (2020). Lipid microbubble-conjugated anti-CD3 and anti-CD28 antibodies (microbubble-based human T cell activator) offer superior long-term expansion of human naive T cells in vitro. Immunohorizons..

[40] Chen G, Yang X, Ko A, Sun X, Gao M, Zhang Y (2017). Sequence and structural analyses reveal distinct and highly diverse human CD8+ TCR repertoires to immunodominant viral antigens. Cell Rep..

[41] Lustig A, Liu HB, Metter EJ, An Y, Swaby MA, Elango P (2017). Telomere shortening, inflammatory cytokines, and anti-cytomegalovirus antibody follow distinct age-associated trajectories in humans. Front Immunol..

