# Supplementary material for: Cytomegalovirus infection reduced CD70 expression, signaling and expansion of viral specific memory CD8+ T cells in healthy human adults
Source: Immun Ageing. 2022 Nov 11;19:54. doi: 10.1186/s12979-022-00307-7 (PMC9650803; doi:10.1186/s12979-022-00307-7)
Supplement: Supplementary file 1 — Additional file 1: Supplementary Figure 1. Expression of different markers inantigen-specific CD8+ cells. Supplementary Figure 2. Percentageof CD28+ antigen-specific TN, TCM and totalCD8+ Tcells. Supplementary Figure 3. ERK1/2 phosphorylation in sorted IAV+and CMV+ TCM cells. Supplementary Figure 4. CD70 expression in Jurkat cells and CD27-CD70 signaling. [file 12979_2022_307_MOESM1_ESM.docx]

**Cytomegalovirus infection reduced CD70 expression, signaling and expansion of viral specific memory CD8^+^ T cells in healthy human adults**

Jian Lu^1†^, Guobing Chen^1,2†^, Arina Sorokina^1^, Thomas Nguyen^1^, Tonya Wallace^1^, Cuong Nguyen^1^, Christopher Dunn^1^, Stephanie Wang^1^, Samantha Ellis^1^, Guixin Shi^3^, Julia McKelvey^4^, Alexei Sharov^5^, Yu-Tsueng Liu^2,3^, Jonathan Schneck^6^, and Nan-ping Weng^1*^

^1^ Laboratory of Molecular Biology and Immunology, National Institute on Aging, NIH, Baltimore, MD, USA

^2^ Current address: Division of Microbiology and Immunology, School of Medicine, Jinan University, 601 Huangpu Ave West, Tianhe District, Guangzhou, China

^3^ Diagnologix LLC, San Diego, CA, USA

^4^ University of California San Diego, La Jolla, CA

^5^ Apheresis unit, Laboratory of Clinical Investigation, National Institute on Aging, NIH, Baltimore, MD, USA

^6^ Laboratory of Genetics and Genomics, National Institute on Aging, NIH, Baltimore, MD, USA

^7^ Department of Pathology, Johns Hopkins University School of Medicine, Baltimore, MD, USA

^†^ These two authors contributed equally.

^*^ Correspondence to Nan-ping Weng ([Wengn@mail.nih.gov](mailto:Wengn@mail.nih.gov))

**Supplemental materials:**

Supplementary Figure 1. Expression of different markers in antigen-specific CD8^+^ cells

Supplementary Figure 2. Percentage of CD28^+^ antigen-specific T_N_, T_CM_ and total CD8^+^ T cells.

Supplementary Figure 3. ERK1/2 phosphorylation in sorted IAV^+^ and CMV^+^ T_CM_ cells

Supplementary Figure 4. CD70 expression in Jurkat cells and CD27-CD70 signaling

Supplementary Table 1. Demographics of study subjects used in the figures (experiments)

Supplementary Table 2. GO pathways identified by GSEA analysis

Supplementary Table 3. Antibody list and information used in flow cytometry analysis

| **A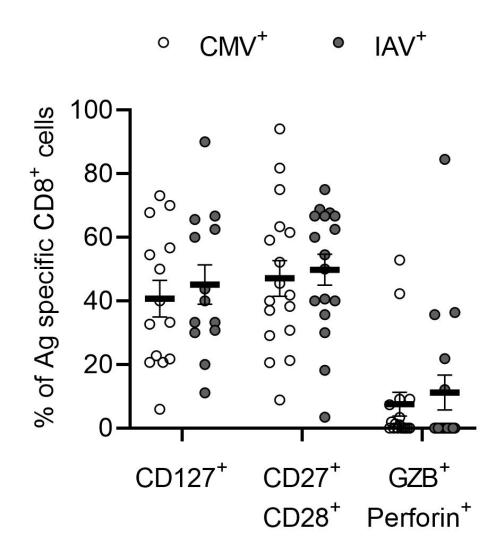** | **B**  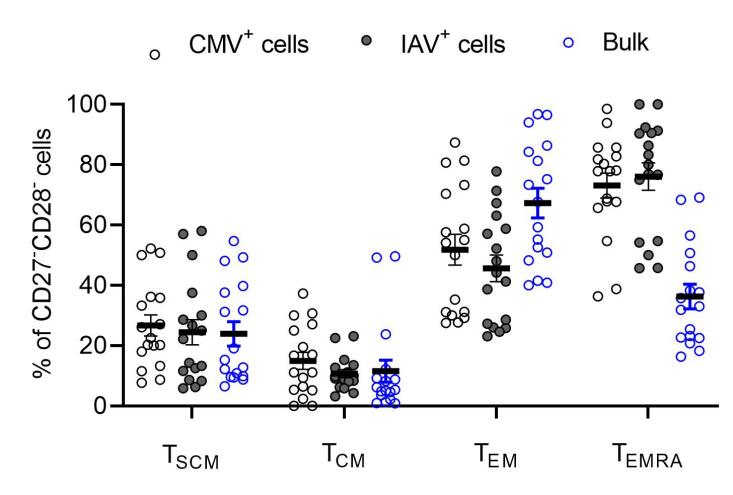 |
| --- | --- |

**Supplementary Figure 1. Expression of different markers in antigen-specific CD8^+^ cells.** (A) Percentages of CD127^+^, CD27^+^CD28^+^, and Granzyme B (GZB)^+^ perforin^+^ cells in CMV-specific and IAV-specific T_N_ CD8^+^ T cell subset from CMV IgG positive young and middle-aged healthy adults (N=17). Data represent the mean values ± standard error of the mean (SEM). (B) Percentages of CD27^-^CD28^-^ CMV-NLV specific, IAV-GIL specific, and total CD8^+^ T cell memory subsets from CMV IgG positive young and middle-aged healthy adults (N=17).

**
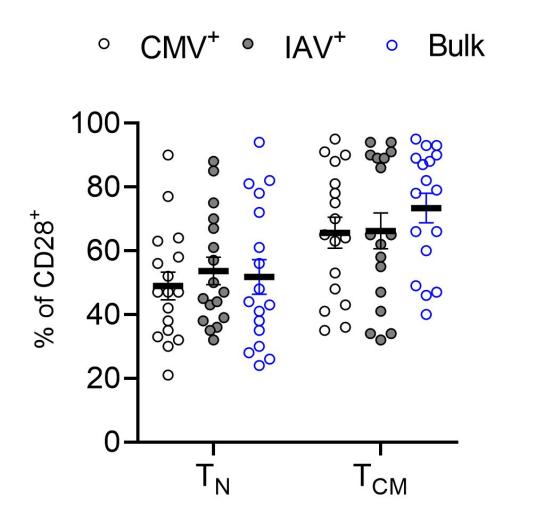
**

**Supplementary Figure 2. Percentages of CD28^+^ and CD27^-^/CD28^-^ in antigen-specific T_N_, T_CM_ and total CD8^+^ T cells.** Percentages of CD28^+^ in CMV-NLV specific, IAV-GIL specific, and total T_N_ and T_CM_ CD8^+^ T cells from CMV IgG positive young and middle-aged healthy adults (N=17). Data represent the mean values ± standard error of the mean (SEM).


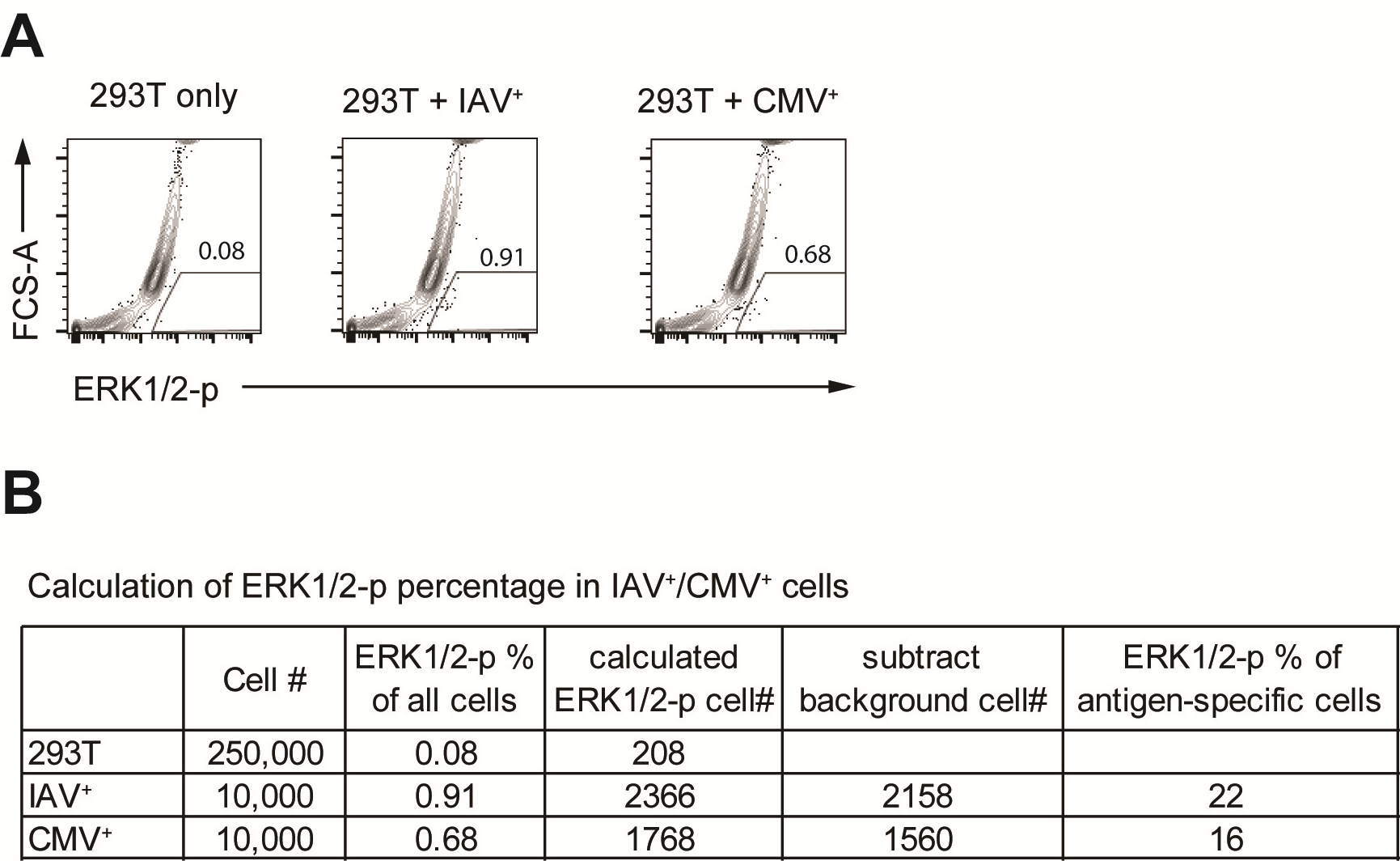


**Supplementary Figure 3. ERK1/2 phosphorylation in sorted IAV^+^ and CMV^+^ T_CM_ cells.** (A) Sorted IAV^+^ and CMV^+^ T_CM_ cells were mixed with 293T cells and stimulated with anti-CD3/CD28 for 15 min and stained with antibody specific for ERK1/2 phosphorylation. Representative flow cytometry results were shown. (B) Example of calculation of ERK1/2 phosphorylation in antigen-specific T_CM_ cells based on the cell numbers of sorted cells and 293T cells used in the mixture.


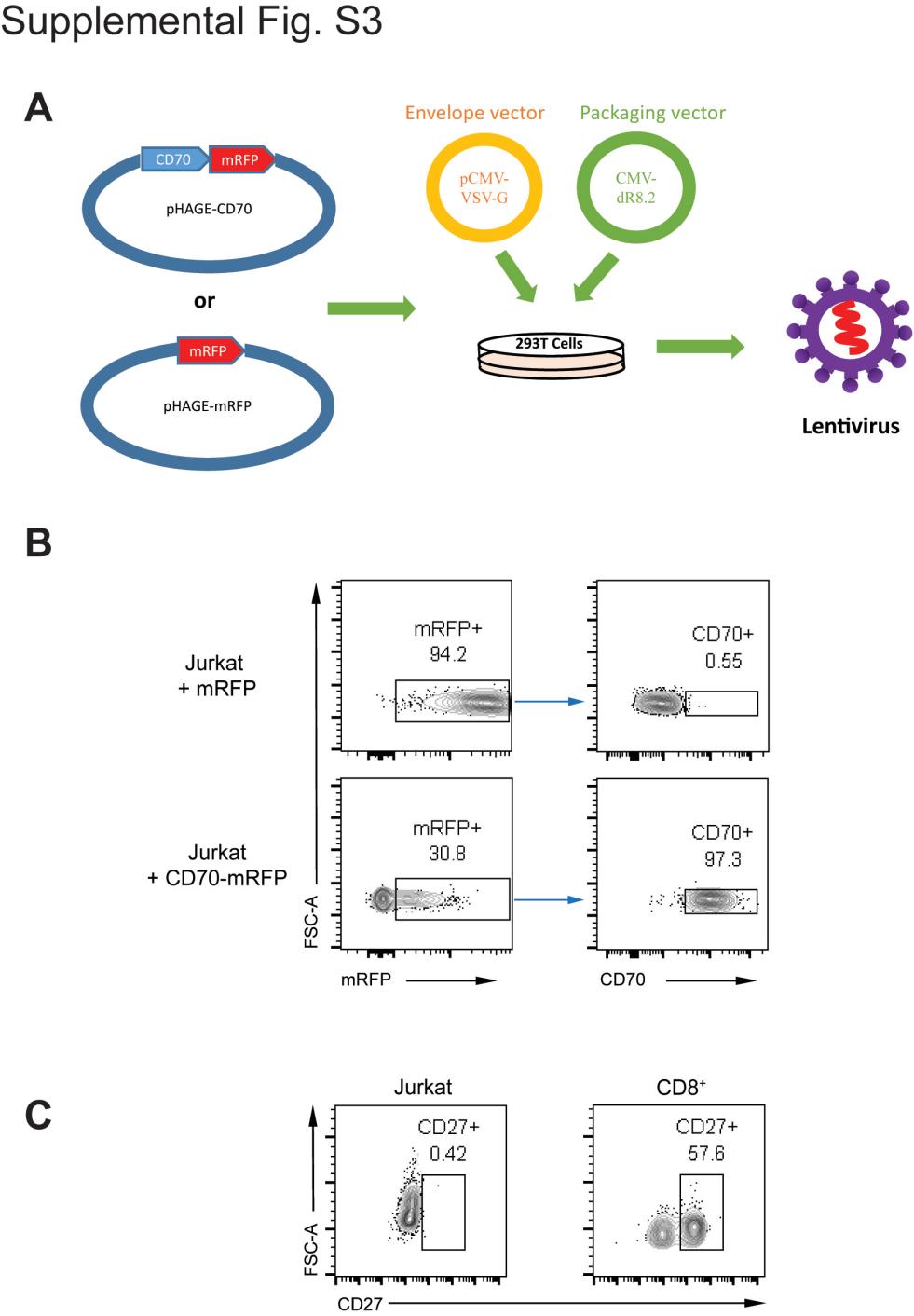


**Supplementary Figure 4. CD70 expression in Jurkat cells and CD27-CD70 signaling.** (A) CD70 gene (NM_001252) was cloned into pHAGE-mRFP vector and lentivirus expressing CD70-mRFP or mRFP alone were prepared from supernatant from 293T cells culture after transduction. (B) Jurkat cells were transduced with lentivirus expressing mRFP or CD70-mRFP and stained with anti-CD70. Jurkat cells do not express CD70, but upon transduction of lentivirus expressing CD70, the majority of mRFP^+^ cells express CD70. (C) Jurkat cells and CD8^+^ T cells were stained with anti-CD27. Jurkat cells do not express CD27.
